# Regional Selection Acting on the *OFD1* Gene Family

**DOI:** 10.1371/journal.pone.0026195

**Published:** 2011-10-14

**Authors:** Ti-Cheng Chang, Jessica L. Klabnik, Wan-Sheng Liu

**Affiliations:** 1 Department of Dairy and Animal Science, The Center for Reproductive Biology and Health, College of Agricultural Sciences, The Pennsylvania State University, University Park, Pennsylvania, United States of America; 2 The Integrative Biosciences Program, Bioinformatics and Genomics Option, The Huck Institute of Life Sciences, The Pennsylvania State University, University Park, Pennsylvania, United States of America; 3 Veterinary and Biomedical Department, College of Agricultural Sciences, The Pennsylvania State University, University Park, Pennsylvania, United States of America; Auburn University, United States of America

## Abstract

The *OFD1* (oral-facial-digital, type 1) gene is implicated in several developmental disorders in humans. The X-linked *OFD1* (*OFD1X*) is conserved in Eutheria. Knowledge about the Y-linked paralog (*OFD1Y*) is limited. In this study, we identified an *OFD1Y* on the bovine Y chromosome, which is expressed differentially from the bovine *OFD1X*. Phylogenetic analysis indicated that: a) the eutherian *OFD1X* and *OFD1Y* were derived from the pair of ancestral autosomes during sex chromosome evolution; b) the autosomal *OFD1* pseudogenes, present in Catarrhini and Murinae, were derived from retropositions of *OFD1X* after the divergence of primates and rodents; and c) the presence of *OFD1Y* in the ampliconic region of the primate Y chromosome is an indication that the expansion of the ampliconic region may initiate from the X-degenerated sequence. In addition, we found that different regions of OFD1/OFD1X/OFD1Y are under differential selection pressures. The C-terminal half of OFD1 is under relaxed selection with an elevated Ka/Ks ratio and clustered positively selected sites, whereas the N-terminal half is under stronger constraints. This study provides some insights into why the *OFD1X* gene causes OFD1 (male-lethal X-linked dominant) and SGBS2 & JSRDs (X-linked recessive) syndromes in humans, and reveals the origin and evolution of the *OFD1* family, which will facilitate further clinical investigation of the OFD1-related syndromes.

## Introduction

The *OFD1* gene (also known as *CXORF5*) has been implicated in several developmental syndromes, including a male-lethal X-linked dominant condition, Oral-Facial-Digital type 1 (OFD1) syndrome [1], X-linked recessive Simpson-Golabi-Behmel syndrome type 2 (SGBS2) [2] and Joubert syndrome and related disorders (JSRDs) [3]. Typical phenotypes of the OFD1 syndrome are malformations of the face, oral cavity, and digits, which often occur with central nervous system (CNS) defects and cystic kidney disease in affected females [4], [5]. The X-linked recessive SGBS2 and JSRD conditions are characterized by severe mental retardation and recurrent respiratory tract infections in both females and males [2], [6]. The human *OFD1* gene maps to the short arm of the human X chromosome (Xp22.2-p22.3), and has been shown to escape X-inactivation [7], [8]. Previous studies revealed that the X-linked *OFD1* (referred to as *OFD1X* in the present study) was expressed differentially at different developmental stages. During early development, *OFD1X* is expressed exclusively in the genital ridges, and later in the nervous system and various craniofacial structures, particularly with a high level in the epithelium lining the oral and nasal cavities [1]. In contrast, *OFD1X* is expressed in all adult tissues during organogenesis [1], [9], [10]. The OFD1X protein is localized in the centrosome and the basal body of primary cilia [11], [12]. Abnormal cilia formation and function are related to deregulation of signal transduction and several types of human disorders, which impact the development of body pattern and the physiology of organ systems [13]–[15]. Further, knockdown of *Ofd1x* in mice has successfully reproduced the features of the human OFD1 syndrome in heterozygous females with increased severity [16]. Distinct from the human ortholog, the mouse *Ofd1x* gene does not escape the X-inactivation, which may be responsible for the observed severity [7], [8]. The *Ofd1x* has been shown to be important not only in organization and assembly of primary cilium, but also the regulation of digit number and identity during limb and skeletal patterning [16]. A recent study in developing zebrafish also suggested that *Ofd1* is essential for normal ciliary motility and function, and is involved in convergent-extension during gastrulation [17]. Thus, the *OFD1* gene family evidently plays an important role in the ciliary formation and function during skeletal development [18].

The OFD1X contains an N-terminal Lis 1 homology (LisH) motif and several coiled-coil (CC) alpha-helical domains in the middle and C-terminus of the proteins [19]. The LisH motif is related to the regulation of microtubule dynamics [20], while the CC domains are important in centrosomal targeting [11]. Different types of *OFD1X* mutations, such as missense, frameshift, nonsense and splicing site mutations, have been observed in patients with OFD1, SGBS2 and JSRD syndromes ([1]–[3], [10], [21]–[24], reviewed in [4], [25]). Most mutations resulted in the loss of CC domains and subsequent deregulation of chromosomal localization [11], whereas the mutations in LisH modified the localization of OFD1X to the Golgi apparatus or nucleus in some cases [4], [20]. Furthermore, *OFD1X* mutations are also correlated with abnormal microtubule dynamics and cell migration as a result of disruption of ciliary localization [19]. Notably, the mutations leading to the OFD1 syndrome have been predominantly present in the N-terminal half (upstream base 1600) of the *OFD1X* gene (83/93, 89%) [4]. The mutations leading to the JSRD and SGBS2 syndromes (three reported to date) are instead present in the C-terminal of the *OFD1X* gene [2], [3]. Unlike the OFD1 syndrome with embryonic male lethality, male patients with JSRD and SGBS2 have a life span up to 30 years old, and carrier females are not affected [3]. Obviously, these syndromes are associated with the unique sites of the mutations as well as the protein structure and function. What is unclear, however, is how and why the mutations from the same X-linked gene (*OFD1X*) can lead to both dominant and recessive conditions.

The *OFD1* orthologs exist in a wide range of species, including mammals, fish, amphibians, and green algae [7], [26]. A genomic analysis identified a pseudogenized, retroposed *OFD1* on the human chromosome 5 [7]. As many as 18 duplicated copies have also been identified on the human Y chromosome, all of which, however, are pseudogenes [7], [9]. Interestingly, an active Y-linked *OFD1* gene (termed *OFD1Y*) in bovine was identified in the present study, which raised fundamental questions as to how does the *OFD1* family evolve and what is the relationship between the sex chromosome-linked *OFD1X* and *OFD1Y?* The objective of this study was to investigate the evolution of the *OFD1* gene family and to examine the role and impacts of selective pressures on *OFD1*. Our findings indicated that the mammalian *OFD1X* and *OFD1Y* were derived from the pair of ancestral autosomes during sex chromosome evolution. The autosomal *OFD1* in primates and rodents was derived from retroposition of *OFD1X*. Furthermore, variable selective pressures along the OFD1/OFD1X/OFD1Y protein were evidenced. The C-terminal half of OFD1 is under relaxed selection, whereas the N-terminal half is under stronger constraints, providing a genetic explanation for the phenotypic variability of *OFD1* related disorders.

## Results

### The *OFD1* gene family

A thorough sequence search retrieved a total of 72 *OFD1* homologs from 31 species in Viridiplantae (including green algae and mosses) and Metazoa (Table 1). The *OFD1* orthologs are present in limited invertebrate lineages, such as sea urchins and tunicates (Table 1). In non-eutherian vertebrates, a single copy *OFD1* ortholog is located on an autosome, i.e. chromosome 9 in zebrafish, 21 in medaka, 1 in chicken and 7 in opossum (Table 1). In Eutheria, the *OFD1X* is well-conserved on the X chromosome for all species investigated to date. In addition to the active *OFD1X*, three major types of *OFD1* pseudogenes were also retrieved (Table 2). The first type includes a single-copy gene located in a conserved syntenic region in primate (on chromosome 5 in human, chimpanzee and orangutan, and chromosome 6 in rhesus monkey). These loci are intronless with long open reading frames (≥ 833 aa). Their promoter regions do not share any homology with the promoter of the *OFD1X* gene and have no promoter signal, suggesting that they are pseudogenes. Similarly, a single-copy, intronless pseudogene was also found in a conserved syntenic region in rodents (on chromosome 2 in mouse and chromosome 3 in rat). However, this region is not syntenic to the one containing the pseudogenized *OFD1* in primates. The intronless gene structure has been considered as a consequence of the retroposition of intron-containing paralogs [27], suggesting that these pseudogenes were derived from the retroposition of the *OFD1X.* The second type includes two lineage-specific pseudogenes with introns, including an X-linked pseudogene in orangutan and an autosomal (chromosome 22) pseudogene in chimpanzee. The third type of pseudogene is present on the eutherian Y chromosome. The human and chimpanzee have at least 18 and 14 copies of *OFD1Y* pseudogenes, respectively, in the ampliconic region of the male-specific region (MSY) (Table 2). The bovine (*Bos Taurus*) Y chromosome (BTAY) contains a single copy *OFD1Y* in the X-degenerated region, which was proposed as a pseudogene in a previous report [28]. The observation of the *OFD1Y* raises questions of whether the X- and Y-linked *OFD1* sequences were once shared during the evolution of the mammalian sex chromosomes, and whether or not there is any active *OFD1Y* gene survived in the mammalian species.

**Table 1:** Gene information of the *OFD1* family.

Organism*	Species	Abbreviation†	Accession no.	Chromosome
(Green algae)	Micromonas sp. RCC299	MICRO	XM_002503105	n.a.
(Green algae)	Chlamydomonas reinhardtii	CHLRE	XM_001691531	n.a.
(Green algae)	Micromonas pusilla	MIRPU	XM_003061119	n.a.
(Mosses)	Physcomitrella patens	PHYPA	XM_001755713	n.a.
(Ciliates)	Tetrahymena thermophila	TETTH	XM_001007171	n.a.
(Placozoans)	Trichoplax adhaerens	TRIAD	XM_002116098	n.a.
(Sea urchins)	Strongylocentrotus purpuratus	STRPU	XM_001178991	n.a.
Florida lancelet	Branchiostoma floridae	BRAFL	XM_002600943	n.a.
(Tunicates)	Ciona intestinalis	CIOIN	ENSCINT00000012613	9
(Hemichordate)	Saccoglossus kowalevskii	SACKO	XM_002733780	n.a.
Zebrafish	Danio rerio	DANRE	NM_001004496	9
Japanese medaka	Oryzias latipes	ORYLA	ENSORLT00000022295	21
Western clawed frog	Xenopus tropicalis	XENTR	XM_002933811	n.a.
Chicken	Gallus gallus	GALGA	XM_416831	1
Three-spined stickleback	Gasterosteus aculeatus	GASAC	ENSGACT00000005222	n.a.
Platypus	Ornithorhynchus anatinus	ORNAN	XM_001515291	n.a.
Gray short-tailed opossum	Monodelphis domestica	MONDO	XM_001381010	7
Horse	Equus caballus	EQUCA	XM_001917181	X
Dog	Canis familiaris	CANFA	XM_537958	X
Cattle	Bos taurus	BOSTA	JN193530	Y
Cattle	Bos taurus	BOSTA	JN193532	X
Norway rat	Rattus norvegicus	RATNO	NM_001106961	X
House mouse	Mus musculus	MUSMU	NM_177429	X
Western European hedgehog	Erinaceus europaeus	ERIEU	ENSEEUT00000009611	n.a.
African savanna elephant	Loxodonta africana	LOXAF	ENSLAFT00000014407	n.a.
Little brown bat	Myotis lucifugus	MYOLU	ENSMLUT00000012689	n.a.
European shrew	Sorex araneus	SORAR	ENSSART00000006423	n.a.
Northern tree shrew	Tupaia belangeri	TUPBE	ENSTBET00000004188	n.a.
Rhesus monkey	Macaca mulatta	MACMU	XM_001098347	X
Bornean orangutan	Pongo pygmaeus	PONPY	ENSPPYT00000023479	X
Chimpanzee	Pan troglodytes	PANTR	XR_022838	X
Human	Homo sapiens	HOMSA	NM_003611	X

*The names of the organisms are given based on the Genbank common name or inherited blast name (in brackets) of the NCBI taxonomy database.

† The abbreviations were used for all analyses.

**Table 2:** Pseudogene information of the *OFD1* family.

Type	Species	Symbol	Chromosome	Coordinates*	Cov (%)†	Idt(%)†	Accession No.
1	Rattus norvegicus	Chr3_RATNOp	3	37679221- 37679189	71.0	87.1	
	Mus musculus	Chr2_MUSMUp	2	55825580- 55825532	47.0	89.8	
	Macaca mulatta	Chr6_MACMUp	6	37048751-37051414	84.0	94.8	
	Pongo pygmaeus	Chr5_ PONPYp	5	38151044-38154241	99.0	95.7	ENSPPYT00000017911
		ChrX_PONPYp	X	13646340- 13652560	26.0	99.9	
	Pan troglodytes	Chr5_ PANTRp	5	77937556-77940573	100.0	94.8	XM_517799
	Homo sapiens	Chr5_HOMSAp	5	37209001-37212697	100.0	94.6	NG_003023
2	Pan troglodytes	ChrY_PANTRp1	Y	7184466- 7201836	68.0	87.8	
		ChrY_PANTRp2	Y	10727522- 10689971	49.0	87.9	
		ChrY_PANTRp3	Y	2759032- 2721228	49.0	87.8	
		ChrY_PANTRp4	Y	3559461- 3597009	49.0	87.8	
		ChrY_PANTRp5	Y	11457924- 11495492	47.0	88.3	
		ChrY_PANTRp6	Y	12093471- 12107199	27.0	87.7	
		ChrY_PANTRp7	Y	1876490- 1888922	27.0	87.8	
		ChrY_PANTRp8	Y	5540507- 5552937	29.0	88.7	
		ChrY_PANTRp9	Y	10530336- 10499144	28.0	87.6	
		ChrY_PANTRp10	Y	3756649- 3788096	28.0	88.8	
		ChrY_PANTRp11	Y	2561590- 2530182	28.0	88.8	
		ChrY_PANTRp12	Y	2172851- 2160421	22.0	87.9	
		ChrY_PANTRp13	Y	11655187- 11655391	10.0	86.9	
		ChrY_PANTRp14	Y	12365121- 12364993	10.0	90.0	
		Chr22_PANTRp	22	15650395- 15650347	63.0	87.9	
	Homo sapiens	ChrY_HOMSAp1	Y	20837254- 20918891	69.0	87.6	
		ChrY_HOMSAp2	Y	20790979- 20744200	61.0	86.6	
		ChrY_HOMSAp3	Y	24760230- 24728791	40.0	87.5	
		ChrY_HOMSAp4	Y	28234642- 28203187	40.0	87.6	
		ChrY_HOMSAp5	Y	25727740- 25759186	41.0	87.9	
		ChrY_HOMSAp6	Y	24118458- 24149894	42.0	87.3	
		ChrY_HOMSAp7	Y	19923003- 19935420	28.0	87.4	
		ChrY_HOMSAp8	Y	21011411- 21029167	24.0	87.9	
		ChrY_HOMSAp9	Y	28043487- 28018078	26.0	88.0	
		ChrY_HOMSAp10	Y	25918892- 25944297	24.0	89.0	
		ChrY_HOMSAp11	Y	20632760- 20244355	23.0	87.1	
		ChrY_HOMSAp12	Y	27842084- 27822990	13.0	88.8	
		ChrY_HOMSAp13	Y	20776169- 20615045	14.0	88.0	
		ChrY_HOMSAp14	Y	8899174- 8908029	13.0	90.8	
		ChrY_HOMSAp15	Y	26120352- 26139444	13.0	88.1	
		ChrY_HOMSAp16	Y	23964359- 23957543	13.0	89.3	
		ChrY_HOMSAp17	Y	20256773- 20256570	6.0	86.8	
		ChrY_HOMSAp18	Y	20882196- 20882222	6.0	96.3	

*The coordinates were derived from the UCSC database.

† The coverage (cov) and identity (idt) were computed based on the alignment with *OFD1X* in each species.

### The discovery of an active *OFD1Y* on the bovine Y chromosome

**Figure 1:**
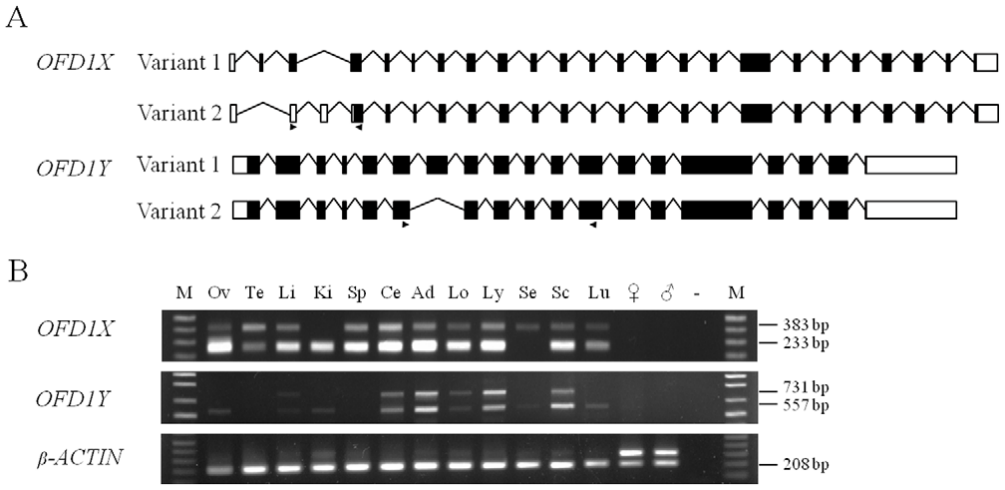
Genomic structures and expression patterns of the bovine *OFD1X* and *OFD1Y*. **A.** Genomic structures of *OFD1X* and *OFD1Y*. Two splicing variants were identified for both *OFD1X* and *OFD1Y* in cattle (see details in Table S1 and S3). PCR primers (arrows) for examining expression patterns by RT-PCR are shown. Introns are not drawn to scale. **B.** Expression patterns of *OFD1X* and *OFD1Y*. The two variants of *OFD1X* are expressed differentially across 12 different bovine tissues: the variant 1 (233 bp) is expressed in all examined tissues except for the semitendinosus, whereas, the variant 2 (383 bp) is undetectable in kidney. Similarly, the variants of *OFD1Y* are expressed differentially across tissues: the variant 1 (731 bp) is detected from liver, cerebellum, adrenal gland, longissimus, lymph node, spinal cord, whereas the variant 2 (557 bp) is expressed in all tissues except for testis and spleen. The expression of the *β-ACTIN* gene was used as a positive control. M, 1Kb DNA ladder; Ov, ovary; Te, testis; Li, liver; Ki, kidney; Sp, spleen; Ce, cerebellum; Ad, adrenal gland; Lo, longisimuss; Ly, lymph node; Se, semitendinosus; Sc, spinal cord; Lu, lung; ♂, bovine male genomic DNA control; ♀, bovine female genomic DNA control; -, negative control (water).

During the analysis of the transcriptome of BTAY, we identified a full-length cDNA sequence (3530 bp, GenBank acc. no. JN193532) of the bovine *OFD1Y* through a deep sequencing of the BTAY-direct selected testis cDNAs [29]. We further confirmed the presence of this Y-linked gene by male-specific PCRs (data not shown), RT-PCRs and an alignment of the cDNA sequence to the position of 294–357 Kb (Table S1) on the BTAY draft sequence (GenBank acc. no. CM001061). The bovine *OFD1Y* is located in MSY between the *ubiquitin specific peptidase 9, Y-linked* (*USP9Y*) and *amelogenin, Y-linked* (*AMELY*) genes, and is approximately 200 Kb away from the pseudoautosomal boundary. Since a previous report suggested that the bovine *OFD1Y* is likely to be a transcribed pseudogene [28], inconsistent with our discovery, it is necessary to further characterize the genomic structure and expression patterns of the bovine *OFD1X* and *OFD1Y* in details. The rapid amplification of the cDNA ends (RACE) and genomic PCR analyses indicated that the bovine *OFD1X* and *OFD1Y* contain 25 and 19 exons, respectively (Fig. 1A), with a sequence similarity of 88% at the nucleotide level and 84% at the protein level. Furthermore, RT-PCR analyses using different combinations of primers across the entire cDNA sequences (Table S2) revealed two splicing variants for *OFD1X* and *OFD1Y*, respectively (Fig 1B). The splicing of *OFD1X* results in the use of an alternative start codon and two different sizes of encoded peptides: 1033 aa in variant 1 (GenBank acc. no. JN193530) and 961 aa in variant 2 (GenBank acc. no. JN193531) (Fig. 1A, Table S3). Similar to *OFD1X*, the bovine *OFD1Y* also underwent splicing leading to two peptides: 875 aa in variant 1 (GenBank acc. no. JN193532) and 817 aa in variant 2 (GenBank acc. no. JN193533) (Fig. 1A, Table S1). The splicing does not impact the domain structure of OFD1X, but it does impact on OFD1Y because the spliced exon 7 (214–271 aa) in OFD1Y is located within one of the CC domains (189-557aa).

To establish the bovine *OFD1X* and *OFD1Y* expression pattern, we performed RT-PCRs across 12 different tissues. The two variants of *OFD1X* are expressed broadly among the majority of tissues examined, while the two variants of *OFD1Y* are expressed at a high level in adrenal gland, lymph node and spinal cord, low or undetectable level in the remaining tissues (Fig. 1B). In addition, the *OFD1X* variant 1 is undetectable in semitendinosus, while the variant 2 is undetectable in kidney (Fig. 1B). The *OFD1Y* variant 2 is detected in more tissues than the variant 1 (Fig. 1B), indicating that the expression of the splicing variants is tissue-specific in cattle. In general, pseudogenes are gene-like sequences, which are lack of splicing signal sequences, transcriptional and translational activities [30]–[32]. The identification of splicing variants, maintenance of an open reading frame with in-frame splicing sites, and different expression levels across tissues indicated that the bovine *OFD1Y* gene is most likely a functional gene, not a transcribed pseudogene as previously suggested [28].

### Phylogenetic analysis of the *OFD1* gene family

**Figure 2:**
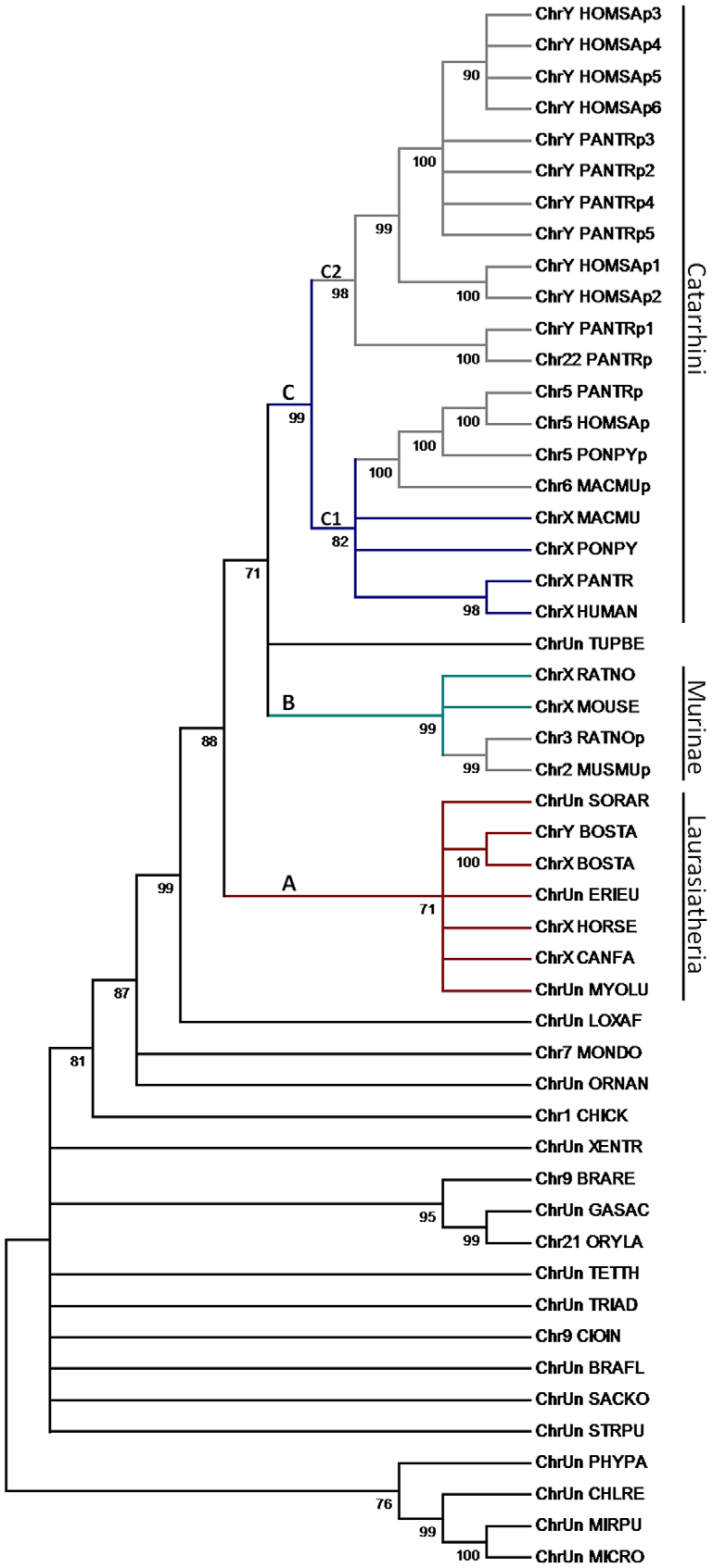
Phylogenetic tree of the *OFD1* gene family. Three major clades are present in the tree: Clade A (red) includes the homologs in Laurasiatheria, in which the bovine *OFD1X* and *OFD1Y* are clustered on one branch; Clade B (green) includes the X-linked *Ofd1x* and retroposed pseudogenes in Murinae; Clade C (blue) includes all the homologs in Catarrhini with two subclades. Subclade C1 includes the primate *OFD1X* and the retroposed autosomal pseudogenes. Subclade C2 includes the largely amplified *OFD1Y* pseudogenes in primates. The branches leading to pseudogenes are in grey. The tree was inferred by the Maximum-likelihood approach and the branches with bootstrap values < 70% were collapsed.

A phylogenetic tree was built using the Maximum-likelihood (ML) method (Fig. 2) [33]. The homologs in Viridiplantae were clustered into one group (Fig. 2). The mammalian homologs were clustered into another large group with a bootstrap value of 88%, within which three clades were present. The first clade (clade A) includes the homologs in Laurasiatheria. The bovine *OFD1Y* gene was grouped with the X paralog with a bootstrap value of 100%. The second clade (clade B) contains the homologs in Rodentia. The autosome-located pseudogenes formed a single cluster indicating a retroposition from *OFD1X* occurred before the divergence of the rodents. The third clade (clade C) comprises all the homologs in Catarrhini with two subclades, C1 and C2. Clade C1 contains the X-linked homologs and the retroposed, autosomal homologs. The clustering pattern suggested that the retroposition of the autosomal homologs in primate occurred before the divergence of primates and after the divergence of primates and rodents. Assuming the divergence time between macaques and orangutans is 30.4 million years ago (MYA) [34], the retroposition of the autosomal homologs in primates was estimated to occur ∼54 MYA (γ_OFD1X_ = 0.000401; γ_OFD1autosome_ = 0.000569; γ_average_ =  0.000458; K_average_ = 0.052500; T_duplication_ = 54.10). Clade C2 comprises the amplified pseudogenes on the Y chromosome in the human and chimpanzee, which still maintain exon-intron structures and are probably derived from duplications of the ancestral *OFD1Y*. The ortholog in treeshrew (*Tupaia belangeri*) was intermingled with the homologs in primates and rodents in the phylogenetic tree. Treeshrews were originally considered insectivores like common shrews (*Sorex araneus*). However, the analyses on skull structure, limbs and genome sequence data have shown that treeshrew is evolutionarily closer to the primate [35], [36]. Therefore, the branching pattern of the *OFD1* gene tree is consistent with the classification. Further, the tree topology revealed that the X-linked pseudogene in orangutan was derived from the duplication of the X-linked counterpart. The autosomal pseudogene on chimpanzee chromosome 22 was derived from the duplication of the Y-linked paralog (Fig. 2).

### Differential selection forces on *OFD1*

**Figure 3:**
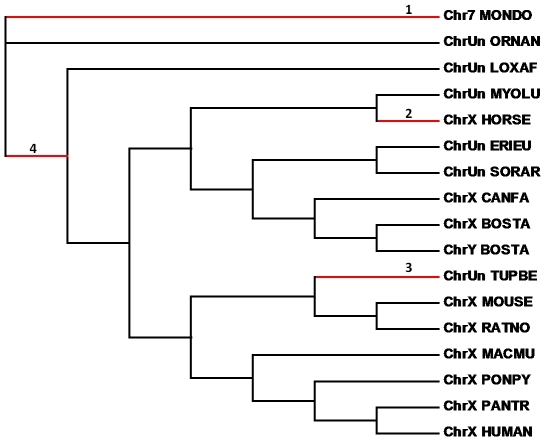
Selection pressures on the mammalian *OFD1*. Four branches of the mammalian *OFD1* tree were identified to be under positive selection (numbered and highlighted in red). The detected sites along each branch are detailed in Table S4.

To study the impact of differential selection forces on the *OFD1* gene family, we first investigated the selective pressures on different lineages and codon positions of the OFD1 protein. To avoid the bias derived from excessively divergent sequences, a dataset containing coding sequences in mammals was used to examine the selection force by the codeml program in PAML [37]. We applied the branch-site models (model A-null v.s. model A) to investigate positive selection [38]. The likelihood ratio tests (LRT) were conducted for each branch (Table S4). Four branches were detected to be under positive selection, including three terminal branches and one internal branch (Fig. 3). The detected terminal branches leading to opossum, horse and treeshrew contain 6, 22, and 4 positively selected sites, respectively (Fig. 3, Table S4). The horse OFD1X contains a high number of selected sites, suggesting it evolved at a fast pace.

The internal branch leading to the eutherians has eight selected sites (Table S4). Six of which were around the CC domains in the C-terminal half of OFD1, one site (76S) was within the LisH domain, and the remaining one was in the N-terminus (Fig. S1). We found that all these positively selected sites were exposed residues, which is in line with the conclusions of previous studies that more exposed residues are less conserved [39], [40].

**Figure 4:**
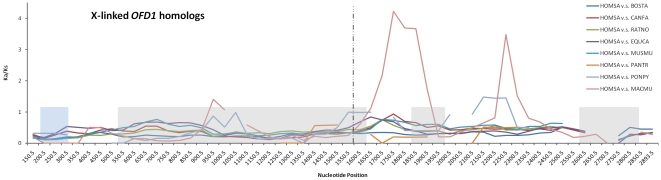
Sliding window analysis of Ka/Ks ratio along the OFD1 protein. Sliding window analysis of Ka/Ks ratio was performed by comparing human OFD1X sequence with other eutherian OFD1X sequences (300 bp window, 50 bp slide). The vertical line represents the position of 1600 nt. Ka/Ks ratio is plotted against the length of the coding region of the mRNAs with a highlighted presentation of protein domains along the x-axis (blue: LisH domain; grey: coiled-coil domains).

To further determine whether different regions of the OFD1 proteins are under distinct selection pressures, we performed a sliding window analysis of Ka/Ks ratio across the *OFD1* coding sequence. The analyses between the human OFD1X with all the other eutherian X-linked orthologs indicated that the Ka/Ks ratio tends to elevate after ∼530 aa (∼1,600 bp), especially in the comparison between the human and macaque OFD1X (Fig. 4). The same trend was observed when comparing pairs of the X-linked and Y-linked/autosomal OFD1 in primates and cattle (Fig. S2). These results were consistent with the clustering pattern of the positively selected sites (see above). Therefore, we divided the protein into two parts (1–529 and 530–1101 aa) and compared their mean and median Ka/Ks ratio, which shows that the values of the N-terminal half are significantly lower than those of the C-terminal half (p<0.001).

## Discussion

### Origin of the mammalian *OFD1* gene family

Although the *OFD1* ortholog is present in vertebrates and green algae, it is not well-conserved in invertebrates [7], [26]. We postulate that the conservation of *OFD1* is associated with the fundamental role of *OFD1* in the ciliary motility [16]. For example, in contrast to human and green algae with motile cilia, the basal bodies in C. elegans are degenerated with singlet microtubules which never form motile cilia [41]. Thus, origin and maintenance of *OFD1* provide necessary gene product for normal ciliary motility and function in specific lineages. The loss of *OFD1*, instead, indicates a replaceable role during a divergent evolution of ciliary formation and function in invertebrates.

The *OFD1* family in mammals was amplified through retroposition and gene duplication. The retroposition of *OFD1X* gave rise to a group of autosomal retro-pseudogenes in primates and rodents, whereas the duplication of *OFD1Y* resulted in a larger group of Y-linked pseudogenes in primates. Compared to the conserved *OFD1X*, the characteristics and functionality of the *OFD1Y* in most mammals is unclear. A major reason for this is the lack of information about the mammalian Y chromosome. To date, only the human, chimpanzee and cattle Y chromosome sequences are publicly available. Two major types of sequences, X-degenerate and ampliconic, are present on MSY [42]. The X-degenerate region harbors mainly single-copy genes/sequences, which share ∼65–95% similarity with the X-counterparts and were derived from a progressive differentiation and degeneration of Y [42]. The ampliconic region comprises mainly Y-specific sequences which underwent multiple duplications and share high intra-chromosomal (Y-to-Y) similarity. The human and chimpanzee *OFD1Y* are largely amplified within the ampliconic region [7] though the amplification mechanism is unknown. It has been found that the two genes, *SEDL* and *RAB9A,* the closest genes to *OFD1X* on the human X chromosome, also had relics on the Y chromosome [7]. These relics are located in the vicinity of the *OFD1Y* and formed *RAB9A–SEDL–OFD1Y* clusters, which are present in the palindromes on the human Y [7]. This observation indicates that the ancestral cluster of *RAB9A–SEDL–OFD1Y* could have first become part of the X-degenerate sequence, then amplified and become part of the ampliconic sequence. This suggests that the expansion of the ampliconic region in primates could be initiated from the X-degenerate region followed by segmental duplications and continuous degeneration. In the present study, we found that the bovine *OFD1Y* is still an active, single-copy gene and resides within the X-degenerate region, suggesting that it was a surviving relic of the ancestral *OFD1* gene during the sex chromosome evolution. We expected that functional *OFD1Y* may be identified in other lineages, especially in Laurasiatheria, when more Y chromosome sequence projects are completed.

### Adaptive evolution and functional modification of *OFD1*

The genes associated with a number of complex diseases have evolved at a faster pace than those not related to diseases, implying a connection between natural selection and disease etiology [43]. Diseases may arise and persist either through a balance between negative selection and mutation, or as a result of adaptation [43]. Therefore, we postulated that the origin of OFD1 syndrome may also be relevant to differential selection pressures on the *OFD1* gene. The selection tests showed that the eutherian *OFD1* homologs were under positive selection, which suggests that they have been subject to functional modifications to acquire lineage-specific roles, a speculation supported by our analyses of *OFD1Y* in cattle. The bovine *OFD1Y* has a different expression pattern from the *OFD1X* (Fig. 1), indicating that the *OFD1Y* may be indispensable in cattle. The maintenance of *OFD1X* and *OFD1Y* in bovine suggested that a continuous selection has acted to modify and refine their function for diverse biological processes. It was also supported by the fact that the duplication patterns of *OFD1* (*OFD1X* and *OFD1Y*) and X-inactivation of *OFD1X* are different between human and mouse [19].

Our sliding window analysis of the Ka/Ks ratio suggested a relaxation of selective pressure in the C-terminal half of the OFD1, which may play a role in the functional adaption of the *OFD1* family and may be associated with the etiology of the OFD1 syndrome. In addition, the distribution pattern of Ka/Ks ratio between the human and macaque *OFD1X* (Fig. 4) was similar to those between the functional *OFD1X* and the autosomal pseudogene in primates (Fig. S2), leading us to speculate the functionality of the macaque *OFD1X* that requires future study.

### The molecular mechanism of the male-lethal X-linked dominant OFD1 syndrome vs. the X-linked recessive SGBS2 and JSRDs syndromes

How could the same *OFD1X* gene result in different sex-linked conditions? The present analyses provide some insights into the molecular mechanism. First, the X-linked dominant vs. recessive conditions are associated with differential selection pressure on different regions of the OFD1 protein. The N-terminal half of OFD1, including LisH motif, is highly constrained among all species studied, suggesting its essentiality and that any mis-sense mutations would lead to a dysfunctional protein. In contrast, selective constraints were relaxed in the C-terminal half of OFD1, and mutations have a higher opportunity to be positively selected as demonstrated by a high proportion (6/8) of positively selected sites detected in this region. In addition, the C-terminal region has a much lower percentage (11%) of OFD1 syndrome related mutations reported. This bias may be explained by the regional selection that may have allowed nucleotide variations to be neutral and persisted in the C-terminal. In addition, the causative genetic variations in C-terminal may lead to more diversified phenotypes due to relaxed selection as shown in the recessive SGBS2 and JSRD cases. Second, the functional *OFD1X* gene in human is under the dosage compensation mechanism. To interpret the dominant and recessive conditions, we use X* to indicate the X chromosome with a mutated *OFD1X*. In the case of the X-linked dominant condition, fetuses with genotypes X*Y will not survive, and patients with X*X will show the syndrome. Thus, clinically, the OFD1 syndrome is defined as a male lethal X-linked dominant condition. As to the X-linked recessive condition, the partially functional mutated OFD1X will allow male patients to survive to a certain age, and one normal copy of OFD1X is enough for normal female development. Therefore, we predict that SGBS2 and JSRDs patients have a genotype of X*Y or X*X* (individual with X*X is normal) (Table 3).

**Table 3:** The *OFD1X* genotypes and phenotypes.

Inheritance	Mutation site (No. of mutations) †	Genotype	Fully functional OFD1X copy no.	Phenotypes
X-dominant		XY	1	Normal
	1–1600 bp (83)	X*Y	0	Embryonic lethality
	1601–3039 bp (9)	X*X (female carrier)	1	OFD1 syndrome
		XX	2	Normal
X-recessive		XY	1	Normal
	1601–3039 bp (3) ‡	X*Y/X*X*	0	JSRD, SGBS2 syndromes
		X*X (female carrier)	1	Normal
		XX	2	Normal

† The A of the start codon (ATG) for human *OFD1X* (acc. no. NM_003611) is referred to as nucleotide 1.The mutation information was derived based on [2]–[4], [25].

‡ The mutations are c.2122-2125dupAAGA [2], c. 2767delG [3], and c. 2841_2847delAAAAGAC [3].

### Conclusions

The eutherian *OFD1* gene family was derived from the pair of ancestral autosomes during sex chromosome evolution, and is under positive selection that may lead to a lineage-dependent modification of *OFD1*. Different regions of OFD1/OFD1X/OFD1Y have experienced differential selective constraints that are stronger at the N-terminal half and more relaxed at the C-terminal half, providing some insights into the genetic mechanism underlying *OFD1*-related syndromes.

## Materials and Methods

### Direct testis cDNA selection and sequencing

The BTAY DNA was isolated by micro-dissection [44]. Library construction, direct testis cDNA selection, and RACE experiments were detailed in Yang *et al.* [29]. The selected cDNAs were sequenced at the National Center for Genome Resources using an Illumina GAIIx.

### RT-PCR

Total RNAs were extracted from 11 tissues (testis, liver, kidney, spleen, cerebellum, adrenal gland, longissimus muscle, lymph node, semitendinosus, spinal cord, and lung) of a 2-year-old bull and an ovarian tissue from a mature cow. These bovine tissues were collected from the slaughterhouse in the Agricultural Experimental Station at the University of Nevada Reno (UNR) following the Biological Agent Use Protocol (UNR permit no. B2005-06). RNAs were then treated with DNase I (Ambion, Austin, TX, USA) and reverse transcribed using Superscript™ III First-Strand Synthesis System (Invitrogen, Carlsbad, CA, USA). RT-PCR was performed in 20 µl containing 10 ng cDNA, 200 µM dNTPs, 1.5 mM MgCl2, 2.5 µM of each primer, 1 unit Taq DNA polymerase (Bioline, Taunton, MA, USA). The PCR conditions were: 94°C for 7 min followed by 35 cycles each of 95°C for 40 sec, 55°C–65°C for 40 sec, 72°C for 40 sec, with a final extension at 72°C for 7 min. Products were resolved on 1.5% agarose gels with ethidium bromide in 1× TAE buffer.

### Identification of the bovine *OFD1X* and *OFD1Y*

Primers were designed to amplify the bovine *OFD1X* gene based on the sequence of NM_001192637 (Table S2). The promoter region of *OFD1X* was predicted using the Eponine [45]. The genomic structure of the bovine *OFD1Y* gene was predicted by the Splign program [46] and confirmed by (RT-)PCR with genomic DNA and testis cDNAs as templates.

### Sequence retrieval and tree building

The human OFD1 (NP_003602.1) was used to query against the NCBI, ENSEMBL and UCSC databases by TBLASTN [47] and Blat [48] to detect homologous regions in the human (Build 37.1), rhesus macaque (Build 1.1), chimpanzee (Build 2.1), mouse (Build 37.1), rat (RGSC v3.4), cattle (Btau 4.0), dog (Build 2.1), horse (EquCab2.0), platypus (Build 1.1), opossum (MonDom5) and in invertebrates (e-value < 1e-5). The retrieved sequences were considered as the OFD1 orthologs when they were the reciprocal best hit of the *OFD1* gene. The sequences that do not have accurate splicing sites, or do not match any EST, or do not have a minimum open reading frame of ≥150 aa were considered pseudogenes. We included the sequences with coverage ≥ 40% of *OFD1X* for tree building. The sequences were pre-aligned using ClustalW [49] based on the codon position and manually adjusted afterwards. The gaps were removed by the Gblocks program [50], [51]. The phylogenetic tree was established using the Maximum Likelihood (ML) and Bayesian Inference approaches [33], [52], which generated a similar tree topology. The reliability of the tree topologies was estimated by the bootstrap test (1000 replicates) [53]. The substitution model used was the General-Time-Reversible model. A discrete Gamma distribution was used to model evolutionary rate differences among sites (parameter  =  1.2846). The rate variation model allowed for some sites to be evolutionarily invariable (0.8202% sites).

### Estimation of the non-synonymous and synonymous nucleotide substitution rates and positive selection test

Since pseudogenes may evolve without selective constraints, a dataset containing only mammalian homologs with coding potential was used to detect positive selection. Similarly, the sequences were first aligned by ClustalW based on codon position and manually adjusted afterwards. Gaps were trimmed using Gblocks. The codeml program in PAML package was used to conduct the selection test. The models used were branch-site models A and A-null. The selected sites were reported when the likelihood ratio test of a specific branch is significant (Bonferroni corrected p-value < 0.05) and posterior probability is > 80% under the Bayes empirical Bayes (BEB) analyses. The sites with posterior probability > 90% were labeled in Fig. S1 and Table S4. The human OFD1 protein, NP_003602.1, was used to predict the OFD1 protein structure by I-TASSER [54]. The confidence score of the protein model is -1.63 and estimated accuracy is 0.52±0.15 TM-score (13.0±4.2 Å (RMSD)). Positively selected sites were mapped to the predicted protein structure. The final result was visualized using Chimera [55]. The solvent accessibility of the sites along the OFD1 protein was predicted using the ACCpro program [56]. The residues with less than 25% relative solvent accessibility were classified as buried residues. Sliding window analysis of Ka and Ks was performed by K-Estimator (300 bp window, 50 bp slide) [57].

## Supporting Information

Figure S1 **3D structure of the OFD1X and the positively selected residues.** Eight sites were detected to be positively selected on the branch leading to eutherians. The sites were mapped to the 3D structure of the human OFD1X protein. The coiled-coil region involved in mediating homo-oligomerization is highlighted in green. Red: posterior possibility (pp) > 0.9; pink: pp>0.5; grey and green: Coiled-coil domains; blue: LisH domain.
(TIF)

Figure S2 **Sliding window Ka/Ks analysis for pairs of the X-linked, Y-linked or autosomal** ***OFD1*** **in cattle and primates**. The analysis was performed by comparing pairs of OFD1 genes in the bovine, macaque, orangutan, chimpanzee (300 bp window, 50 bp slide). Ka/Ks ratio is plotted against the length of the coding region of the mRNAs.
(TIF)

Table S1 **The genomic structure of the bovine** ***OFD1Y*** **.**
(DOC)

Table S2 **Sequences of primers designed for PCR and RT-PCR.**
(DOC)

Table S3 **The genomic structure of the bovine** ***OFD1X*** **.**
(DOC)

Table S4 **Positively selected branches and sites in the mammalian** ***OFD1*** **homologs.**
(DOC)
