# Supplementary figures and images for: Regional Selection Acting on the OFD1 Gene Family
Source: PLoS One. 2011 Oct 14;6(10):e26195. doi: 10.1371/journal.pone.0026195 (PMC3193505; doi:10.1371/journal.pone.0026195)

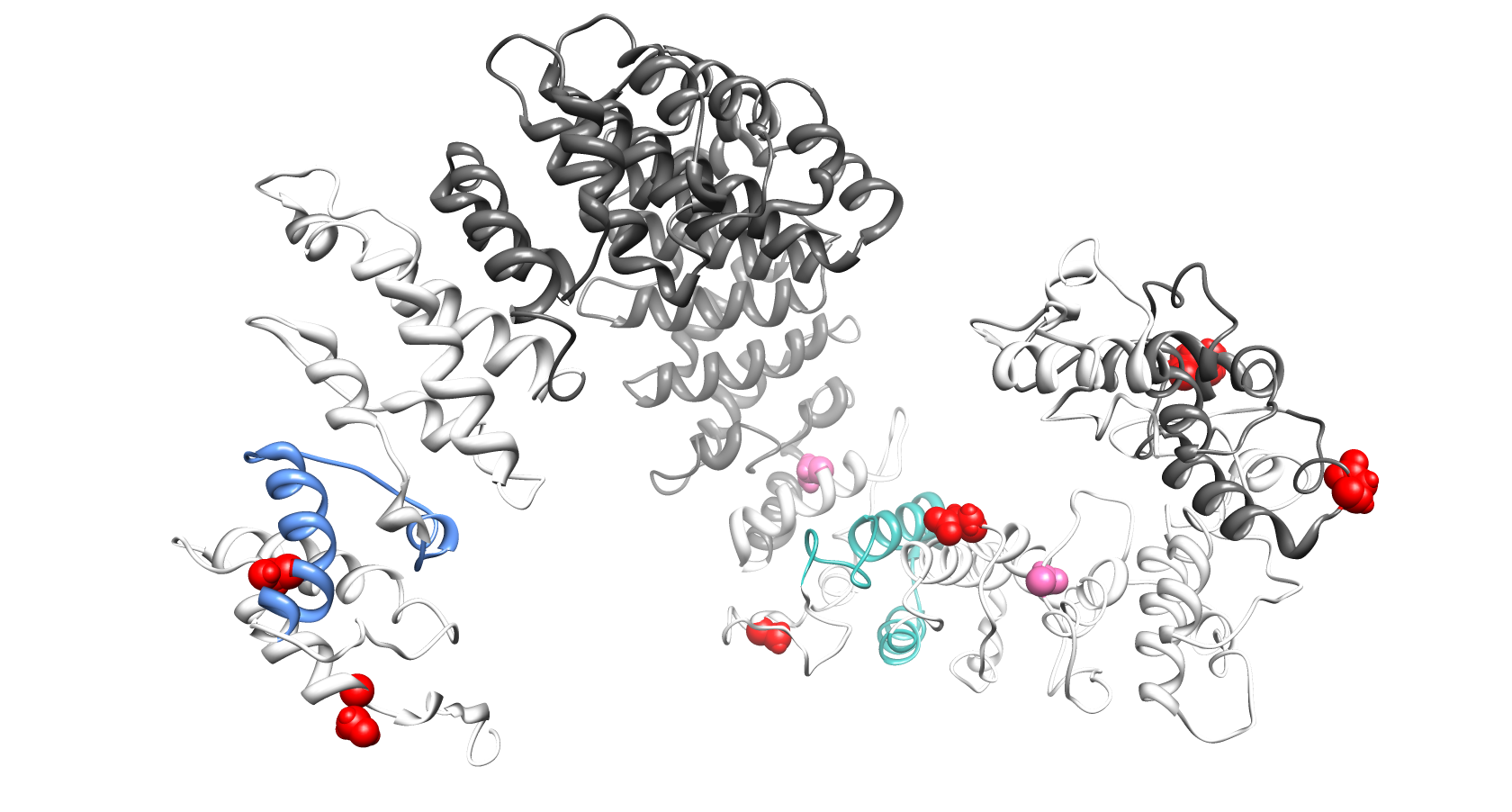

Supplement: Figure S1 — 3D structure of the OFD1X and the positively selected residues. Eight sites were detected to be positively selected on the branch leading to eutherians. The sites were mapped to the 3D structure of the human OFD1X protein. The coiled-coil region involved in mediating homo-oligomerization is highlighted in green. Red: posterior possibility (pp) > 0.9; pink: pp>0.5; grey and green: Coiled-coil domains; blue: LisH domain. (TIF) [file pone.0026195.s001.tif]

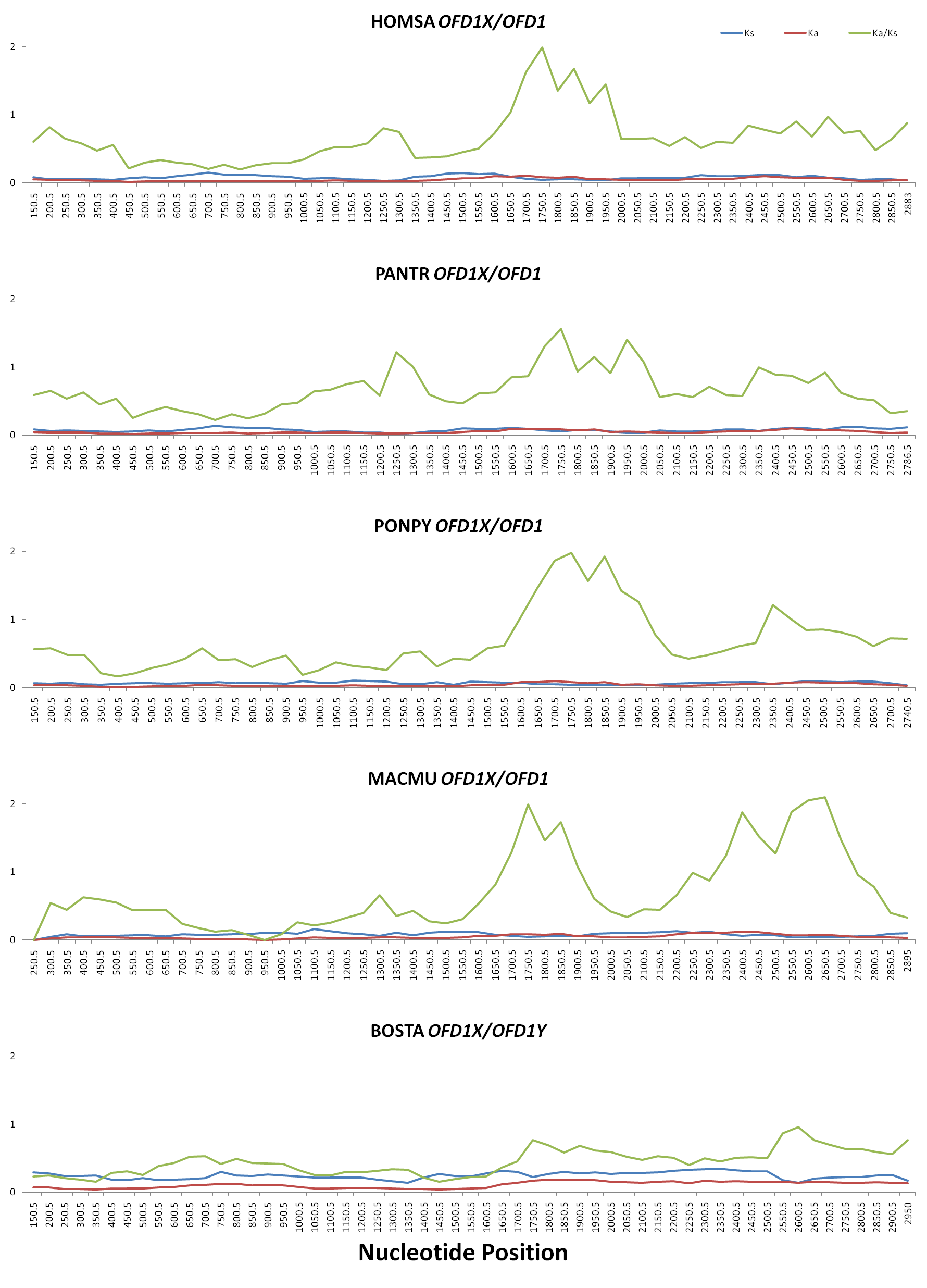

Supplement: Figure S2 — Sliding window Ka/Ks analysis for pairs of the X-linked, Y-linked or autosomal OFD1 in cattle and primates. The analysis was performed by comparing pairs of OFD1 genes in the bovine, macaque, orangutan, chimpanzee (300 bp window, 50 bp slide). Ka/Ks ratio is plotted against the length of the coding region of the mRNAs. (TIF) [file pone.0026195.s002.tif]
