# Supplementary material for: Regional Selection Acting on the OFD1 Gene Family
Source: PLoS One. 2011 Oct 14;6(10):e26195. doi: 10.1371/journal.pone.0026195 (PMC3193505; doi:10.1371/journal.pone.0026195)
Supplement: Table S1 — The genomic structure of the bovine OFD1Y . (DOC) [file pone.0026195.s003.doc]

Table S1***.*** The genomic structure of the bovine *OFD1Y*.

| Exon | Y-chr assembly | Length | *OFD1Y*  start | *OFD1Y*  end | Y-chr  start | Y-chr  end | Splicing type |
| --- | --- | --- | --- | --- | --- | --- | --- |
| 1 | CM001061 | 226 | 1 (134)^Δ^ | 226 | 294019 | 294244 | <exon>GT |
| 2 | CM001061 | 201 | 227 | 427 | 300174 | 300374 | AG<exon>GT |
| 3 | CM001061 | 69 | 428 | 496 | 302761 | 302829 | AG<exon>GT |
| 4 | CM001061 | 31 | 497 | 527 | 302918 | 302948 | AG<exon>GT |
| 5 | CM001061 | 108 | 528 | 635 | 311987 | 312094 | AG<exon>GT |
| 6 | CM001061 | 137 | 636 | 772 | 313612 | 313748 | AG<exon>GT |
| 7^*^ | CM001061 | 174 | 773 | 946 | 314061 | 314234 | AG<exon>GT |
| 8 | CM001061 | 107 | 947 | 1053 | 319329 | 319435 | AG<exon>GT |
| 9 | CM001061 | 117 | 1054 | 1170 | 321386 | 321502 | AG<exon>GT |
| 10 | CM001061 | 74 | 1171 | 1244 | 323839 | 323912 | AG<exon>GT |
| 11 | CM001061 | 92 | 1245 | 1336 | 325175 | 325266 | AG<exon>GT |
| 12 | CM001061 | 190 | 1337 | 1526 | 326281 | 326470 | AG<exon>GT |
| 13 | CM001061 | 131 | 1527 | 1657 | 329107 | 329237 | AG<exon>GT |
| 14 | CM001061 | 112 | 1658 | 1769 | 329760 | 329871 | AG<exon>GT |
| 15 | CM001061 | 597 | 1770 | 2366 | 342594 | 343190 | AG<exon>GT |
| 16 | CM001061 | 121 | 2367 | 2487 | 343665 | 343785 | AG<exon>GT |
| 17 | CM001061 | 101 | 2488 | 2588 | 348625 | 348725 | AG<exon>GT |
| 18 | CM001061 | 158 | 2589 | 2746 | 356062 | 356219 | AG<exon>GT |
| 19 | CM001061 | 784 | 2747 | (2758)3530 ^†^ | 356844 | 357627 | AG<exon>TA |

*The 7^th^ exon is spliced out in variant 2.

^†^ The start and end positions of CDS.
