# Supplementary material for: Regional Selection Acting on the OFD1 Gene Family
Source: PLoS One. 2011 Oct 14;6(10):e26195. doi: 10.1371/journal.pone.0026195 (PMC3193505; doi:10.1371/journal.pone.0026195)
Supplement: Table S2 — Sequences of primers designed for PCR and RT-PCR. (DOC) [file pone.0026195.s004.doc]

Table S2. Sequences of primers designed for PCR and RT-PCR*.*

| Gene | Primer name | Primer | Position* |
| --- | --- | --- | --- |
| OFD1X | M452F | CTCAACTTCCGTAGGGACGC | - |
| OFD1X | 77F^†^ | TCTCCTGGTCCATCATGTTC | - |
| OFD1X | 304F | GTGGCCGATCACTTGCAGAG | - |
| OFD1X | 328R^†^ | CACATCTCTGCAAGTGATCG | - |
| OFD1X | 1312R | CAGACTCCAATTCAAGCTCG | - |
| OFD1X | 1345F | AACCACATGCTAAGCGAG | - |
| OFD1X | 1503R | AGACGGCTGAGCTATGCGG | - |
| OFD1X | 1826R | TCGCCATCACGAGGTTCCAC | - |
| OFD1X | 2369R | TCAGGACAGGTGGTGGTGAC | - |
| OFD1X | 3117R | GGGCAGCCAACATGATTACC | - |
| OFD1X | 3393F | CTCCCTTTTCGAACTTTCAG | - |
| OFD1X | 3500R | TTCAGGGACAAACAATAAGG | - |
| OFD1Y | F | ACTTGTTTCCTAACCCTAACC | 72107 |
| OFD1Y | R | GCCACATCTCTGTAAGTGAC | 65806 |
| OFD1Y | F1 | TCCAACTTTCTAGTAGCCAG | 65844 |
| OFD1Y | F2 | CTTTCAAGAGGTTAACTGTG | 36317 |
| OFD1Y | R2^‡^ | TTCAGTAGTGAATAGTTGTC | 39745 |
| OFD1Y | F3 | GAAGCTCACCTACCCTCTAC | 23190 |
| OFD1Y | R3 | CAGTTGTGATTTTAAATTGG | 36271 |
| OFD1Y | F4 | GATGGAGTGTGCCAAGGCTG | 15760 |
| OFD1Y | R4 | TCCAGCATTTCAAGTTCTCC | 9916 |
| OFD1Y | F5^‡^ | GAAATCAAGTTAAATGAATG | 52443 |
| OFD1Y | R5 | AAGACAGTGCTTCTGGTTGC | 23492 |
| OFD1Y | F6 | AAACTCTGCAGAGAGAGTGG | 9957 |
| OFD1Y | R6 | ACAGAGAATTCACAGGCCAC | 8139 |
| OFD1Y | F7 | TGCTGGTGCTTGTCTTCAAC | 54283 |
| OFD1Y | R7 | TTCGGCAACATTACCACCAG | 6673 |
| OFD1Y | F8 | GTTGCACTGCCATCTTCTCC | 22525 |
| OFD1Y | F9 | GGCAGGAAGAAGAGCAGAGG | 9992 |
| β -Actin | β -ActinF | CTGGACTTCGAGCAGGAGAT | - |
| β -Actin | β -ActinR | GGATGTCGACGTCACACTTC | - |

*The positions are defined based on the *OFD1Y*-located Y-BAC, AC216981.4.

^†^ The primers used in RT-PCR for examining the expression pattern of *OFD1X*.

^‡^ The primers used in RT-PCR for examining the expression pattern of *OFD1Y*.
