# Supplementary material for: Regional Selection Acting on the OFD1 Gene Family
Source: PLoS One. 2011 Oct 14;6(10):e26195. doi: 10.1371/journal.pone.0026195 (PMC3193505; doi:10.1371/journal.pone.0026195)
Supplement: Table S3 — The genomic structure of the bovine OFD1X . (DOC) [file pone.0026195.s005.doc]

Table S3. The genomic structure of the bovine *OFD1X*.

| Exon | X-contig | Length | OFD1X  start | OFD1X  end | X-contig  start | X-contig  end | Splicing type |
| --- | --- | --- | --- | --- | --- | --- | --- |
| 1 | NW_001508851 | 116 | 1 | 116 | 520932 | 520817 | <exon>GT |
| 2* | NW_001508851 | 57 | 117(117) ^‡^ | 173 | 520695 | 520639 | TG<exon>GT |
| 3 | NW_001508851 | 126 | 174 | 299 | 520543 | 520418 | AG<exon>GT |
| 4 ^†^ | NW_001508851 | 150 | 300 | 449 | 511606 | 511457 | AG<exon>GT |
| 5 | NW_001508851 | 201 | 450(483) ^¶^ | 650 | 511286 | 511086 | AG<exon>GT |
| 6 | NW_001508851 | 69 | 651 | 719 | 507740 | 507672 | AG<exon>GT |
| 7 | NW_001508851 | 31 | 720 | 750 | 507583 | 507553 | AG<exon>GT |
| 8 | NW_001508851 | 108 | 751 | 858 | 500173 | 500066 | AG<exon>GT |
| 9 | NW_001508851 | 137 | 859 | 995 | 498542 | 498406 | AG<exon>GT |
| 10 | NW_001508851 | 174 | 996 | 1169 | 498088 | 497915 | AG<exon>GT |
| 11 | NW_001508851 | 107 | 1170 | 1276 | 491883 | 491777 | AG<exon>GT |
| 12 | NW_001508851 | 120 | 1277 | 1396 | 489983 | 489864 | AG<exon>GT |
| 13 | NW_001508851 | 74 | 1397 | 1470 | 488198 | 488125 | AG<exon>GT |
| 14 | NW_001508851 | 92 | 1471 | 1562 | 486892 | 486801 | AG<exon>GT |
| 15 | NW_001508851 | 190 | 1563 | 1752 | 485792 | 485603 | AG<exon>GT |
| 16 | NW_001508851 | 131 | 1753 | 1883 | 482906 | 482776 | AG<exon>GT |
| 17 | NW_001508851 | 112 | 1884 | 1995 | 482245 | 482134 | AG<exon>GT |
| 18 | NW_001508851 | 603 | 1996 | 2598 | 472434 | 471832 | AG<exon>GT |
| 19 | NW_001508851 | 118 | 2599 | 2716 | 471365 | 471248 | AG<exon>GT |
| 20 | NW_001508851 | 101 | 2717 | 2817 | 469829 | 469729 | AG<exon>GT |
| 21 | NW_001508851 | 120 | 2818 | 2937 | 463941 | 463822 | AG<exon>GT |
| 22 | NW_001508851 | 158 | 2938 | 3095 | 461527 | 461370 | AG<exon>GT |
| 23 | NW_001508851 | 159 | 3096 | 3254 | 460884 | 460726 | AG<exon>GT |
| 24 | NW_001508851 | 71 | 3255 | 3325 | 460186 | 460116 | AG<exon>GT |
| 25 | NW_001508851 | 476 | 3326 | (3365)3801 ^‡¶^ | 459841 | 459366 | AG<exon>GT |

^*^ The 2^nd^ exon is spliced out from variant 2.

^†^ The 4^th^ exon is spliced out from variant 1.

^‡^ The start and end positions of CDS in variant 1.

^¶^ The start and end positions of CDS in variant 2.
