# Supplementary material for: Regional Selection Acting on the OFD1 Gene Family
Source: PLoS One. 2011 Oct 14;6(10):e26195. doi: 10.1371/journal.pone.0026195 (PMC3193505; doi:10.1371/journal.pone.0026195)
Supplement: Table S4 — Positively selected branches and sites in the mammalian OFD1 homologs. (DOC) [file pone.0026195.s006.doc]

***Table S4. Positively selected branches and sites in the mammalian OFD1 homologs.***

| Model | Branch^*^ | np | lnl | 2lnl | df | p-value^†^ | Site^‡^ |
| --- | --- | --- | --- | --- | --- | --- | --- |
| a | 1 | 42 | -24301.60 | 21.71 | 1 | 9.83E-05 | **284A**,601A,758S,767P,***774S***,775P |
| a null | 1 | 41 | -24312.46 | - | - | - | - |
| a | 2 | 42 | -24262.06 | 69.37 | 1 | 2.53E-15 | ***223K***,227I,**229K**,***235K***,**240K**,***241E***,  ***245F***,246Q,***248D***,***250E***,252A,**257S**,**259A**,262L,***264E***,***271I***,**272H**,***273K***,  ***274H***,**275Q**,***647K***,**668K** |
| a null | 2 | 41 | -24296.75 | - | - | - | - |
| a | 3 | 42 | -24305.71 | 19.10 | 1 | 3.85E-04 | **620S**,622D,658K,**700E**,***948K*** |
| a null | 3 | 41 | -24315.26 | - | - | - | - |
| a | 4 | 42 | -24298.91 | 27.29 | 1 | 5.43E-06 | **15S**,**76S**, 569S, **601A**, **708E**,758S,**875E**,***897R*** |
| a null | 4 | 41 | -24312.56 | - | - | - | - |

^*^ The corresponding branches are shown in Fig. 3.

^†^ The p-values are adjusted by the Bonferroni correction method.

^‡^ The site positions are annotated based on the human *OFD1X*. The site with a posterior probability > 0.8 is listed. The site with a posterior probability > 0.9 under BEB analyses is in bold; the site with a posterior probability > 0.95 under BEB analyses is in bold italic.
